# Observation of a Nearly Field-Independent Ferromagnetic Resonance Frequency in an Epitaxial Co_25_Fe_75_ Thin Film

**DOI:** 10.3390/ma19173571

**Published:** 2026-08-22

**Authors:** Aleksandra Napierała-Batygolska, Piotr Graczyk, Adam Krysztofik

**Affiliations:** 1 Faculty of Physics and Astronomy, Adam Mickiewicz University Poznan, Uniwersytetu Poznańskiego 2, 61-614 Poznań, Poland; 2 Institute of Molecular Physics, Polish Academy of Sciences, Smoluchowskiego 17, 60-179 Poznań, Poland; piotr.graczyk@ifmpan.poznan.pl

**Keywords:** Co–Fe alloys, epitaxial thin films, magnetocrystalline anisotropy, ferromagnetic resonance, VNA-FMR, magnetization dynamics

## Abstract

We investigate the dynamic magnetic properties of an epitaxial Co_25_Fe_75_ thin film grown on a MgAl_2_O_4_ (001) substrate using broadband ferromagnetic resonance (VNA-FMR). The film exhibits a pronounced fourfold symmetry of the resonance field, characteristic of cubic magnetocrystalline anisotropy. By combining broadband and angular-dependent FMR measurements, we determined a spectroscopic *g*-factor of 2.083 ± 0.017, an effective magnetization of 1655 ± 31 kA/m, and a cubic magnetocrystalline anisotropy field of 28.25 ± 0.22 mT. Beyond the expected angular dependence of the resonance field, we experimentally demonstrated a pronounced flattening of the frequency versus magnetic field dependence for magnetic field direction located between the principal crystallographic axes. The effect, predicted by conventional ferromagnetic resonance theory but not previously investigated in detail, originates from the equilibrium rotation of the magnetization and is quantitatively described within the Stoner–Wohlfarth framework. For $\phi_{H}$ = 34°, the resonance frequency remained nearly constant over the magnetic field interval from 6.8 to 26.2 mT at room temperature. A comparison with other (001)-oriented epitaxial magnetic films revealed that similar frequency plateaus can occur over frequencies ranging from 0.9 to 12.35 GHz and over magnetic field intervals from 0.5 to 63 mT. These findings establish a route toward microwave devices that are insensitive to fluctuations in the applied magnetic field and motivate further studies of spin-wave dynamics in this regime.

## 1. Introduction

Co–Fe alloys have attracted considerable scientific attention owing to their outstanding magnetic properties [1,2,3]. Among the 3d transition-metal alloys, they exhibit some of the highest saturation magnetizations, as predicted by the Slater–Pauling model, while simultaneously combining low magnetic damping with a high magnetocrystalline anisotropy [2,3,4,5,6,7,8,9,10,11]. These properties have motivated extensive research ranging from first-principles calculations of their electronic and magnetic structure to applications in magnetic sensing, spin-transfer-torque devices, magnonics, water splitting, and energy-efficient microwave technologies [2,3,12,13,14,15,16,17,18].

In particular, Co–Fe alloys have emerged as model systems for investigating the interplay between crystal symmetry and magnetic anisotropy [2,7,8]. When grown epitaxially, the cubic crystal structure gives rise to a pronounced fourfold magnetic anisotropy, which strongly influences their static and dynamic magnetic response [4,8]. Ferromagnetic resonance (FMR) is a well-established technique for investigating these effects in thin film systems, allowing determination of the Landé g-factor, magnetic anisotropy, and effective magnetization [4,19,20,21,22,23,24,25].

In this work, we investigate the dynamic magnetic properties of an epitaxial Co_25_Fe_75_ thin film, corresponding to the composition near the maximum of the Slater–Pauling curve [2]. Compared to other Co–Fe compositions, films of Co_25_Fe_75_ exhibit the highest values of saturation magnetization and the lowest values of magnetization damping [1,2,26,27]. The film was grown on a MgAl_2_O_4_ substrate, which provides excellent lattice matching for epitaxial growth. The Co_25_Fe_75_ unit cell ($a_{CoFe}=$ 0.287 nm) was rotated by 45° with respect to the MgAl_2_O_4_ lattice ($a_{MAO}$ = 0.808 nm), resulting in the supercell relation $\sqrt{2}a_{MAO}\approx 4a_{CoFe}$ and a lattice mismatch of only 0.4%. Such a small mismatch minimizes epitaxial strain and reduces the density of misfit dislocations, thereby enabling the growth of high-quality epitaxial films.

For epitaxial (001)-oriented Co_25_Fe_75_ films, the cubic crystal symmetry is expected to manifest itself through a fourfold angular dependence of the resonance field. Beyond this well-known feature, standard FMR modeling predicts a pronounced flattening of the frequency vs. magnetic field dependence for the magnetic field direction located between the principal crystallographic axes. Although this behavior naturally emerges from the theoretical description, to the best of our knowledge, it has not yet been experimentally demonstrated. The primary objectives of this work are therefore twofold: first, to determine the magnetic anisotropy and dynamic magnetic parameters of an epitaxial Co_25_Fe_75_/MgAl_2_O_4_ film using broadband VNA-FMR measurements; and second, to experimentally verify and analyze the predicted frequency plateau.

The remainder of the article is organized as follows. Section 2 describes the sample preparation and the experimental methods. Section 3 presents the theoretical framework and data-analysis methodology. Section 4.1 discusses magnetostatic properties, with particular emphasis on the magnetocrystalline anisotropy and saturation magnetization of the Co_25_Fe_75_ film. Section 4.2 presents the ferromagnetic resonance results obtained in field-swept mode at high frequencies. Section 4.3 focuses on frequency-swept measurements in the low-field regime, and in particular, on the observed flattening of the frequency vs. magnetic field dependence. Section 4.4 provides a broader comparison with other (001)-oriented epitaxial thin films based on the literature data. Finally, Section 5 summarizes the main findings of this work.

## 2. Materials and Methods

The sample preparation procedure was as follows. The (001)-oriented MgAl_2_O_4_ substrate was cleaned by ultrasonic agitation in acetone and isopropanol to remove surface contaminants. The films were deposited by magnetron sputtering with a base pressure of approximately 5·10^−8^ mbar using an alloy target of Co_25_Fe_75_ and a Au target. A 7 nm-thick Co_25_Fe_75_ film was deposited at 350 °C. To prevent oxidation, a 5 nm-thick Au capping layer was deposited after the temperature had decreased below 100 °C. The nominal thicknesses were further confirmed using X-ray diffraction measurements (Appendix A).

Magnetization reversal curves were recorded using a home-built vibrating sample magnetometer (VSM). The measurements were performed at room temperature. The accuracy of the in-plane sample alignment with respect to the direction of the external magnetic field was estimated to be ±2°.

Broadband ferromagnetic resonance measurements using a vector network analyzer (VNA-FMR) were performed at room temperature. The sample was placed on a coplanar waveguide. The external magnetic field was applied in the plane of the sample and swept at a constant excitation frequency. The measurements were carried out over the frequency range from 8 to 40 GHz, with the magnetic field applied along the easy magnetization axis of the Co_25_Fe_75_ film. The complex transmission parameter, S_21_, was recorded to obtain absorption spectra, which were subsequently fitted with a Lorentzian function to determine the resonance field.

Angle-resolved in-plane VNA-FMR measurements were also performed in field-swept mode at fixed excitation frequencies of 10 and 20 GHz by rotating the sample with respect to the external magnetic field. The angular step size was 10°, and the accuracy of the angular alignment was estimated to be ±2°.

For low magnetic fields ranging from −60 to 60 mT, frequency-swept VNA-FMR measurements were performed. The excitation frequency was varied from 2 to 12 GHz. To improve the signal-to-noise ratio, a reference measurement was taken at an external magnetic field of −120 mT, and the differential signal was calculated as $∆S_{21}=S_{21}^{meas}-S_{21}^{Ref}$ [28].

## 3. Theoretical Background

For a (001)-oriented epitaxial Co_25_Fe_75_ film deposited on a MgAl_2_O_4_ substrate, we considered the total free energy density F that includes the Zeeman energy $F_{Zee}$, the demagnetization energy $F_{d}$, the magnetocrystalline cubic anisotropy energy $F_{4}$, and the uniaxial out-of-plane anisotropy energy $F_{2⊥}$:

(1) $$F=F_{Zee}+F_{d}+F_{4}+F_{2⊥},$$

(2) $$F_{Zee}=-\mu_{0}HM_{s}\left(sin\theta_{M}\;sin\theta_{H}\;cos\left(\phi_{H}-\phi_{M}\right)+cos\theta_{M}\;cos\theta_{H}\right),$$

(3) $$F_{d}=\frac{1}{2}\mu_{0}M_{s}^{2}\;{cos}^{2}\theta_{M},$$

(4) $$F_{4}=\frac{1}{2}\mu_{0}H_{4}M_{s}\;{sin}^{2}\theta_{M}\left({cos}^{2}\theta_{M}+{cos}^{2}\phi_{M}{sin}^{2}\theta_{M}{sin}^{2}\phi_{M}\right),$$

(5) $$F_{2⊥}=-\frac{1}{2}\mu_{0}H_{2⊥}M_{s}\;{cos}^{2}\theta_{M}.$$

**Figure 1:**
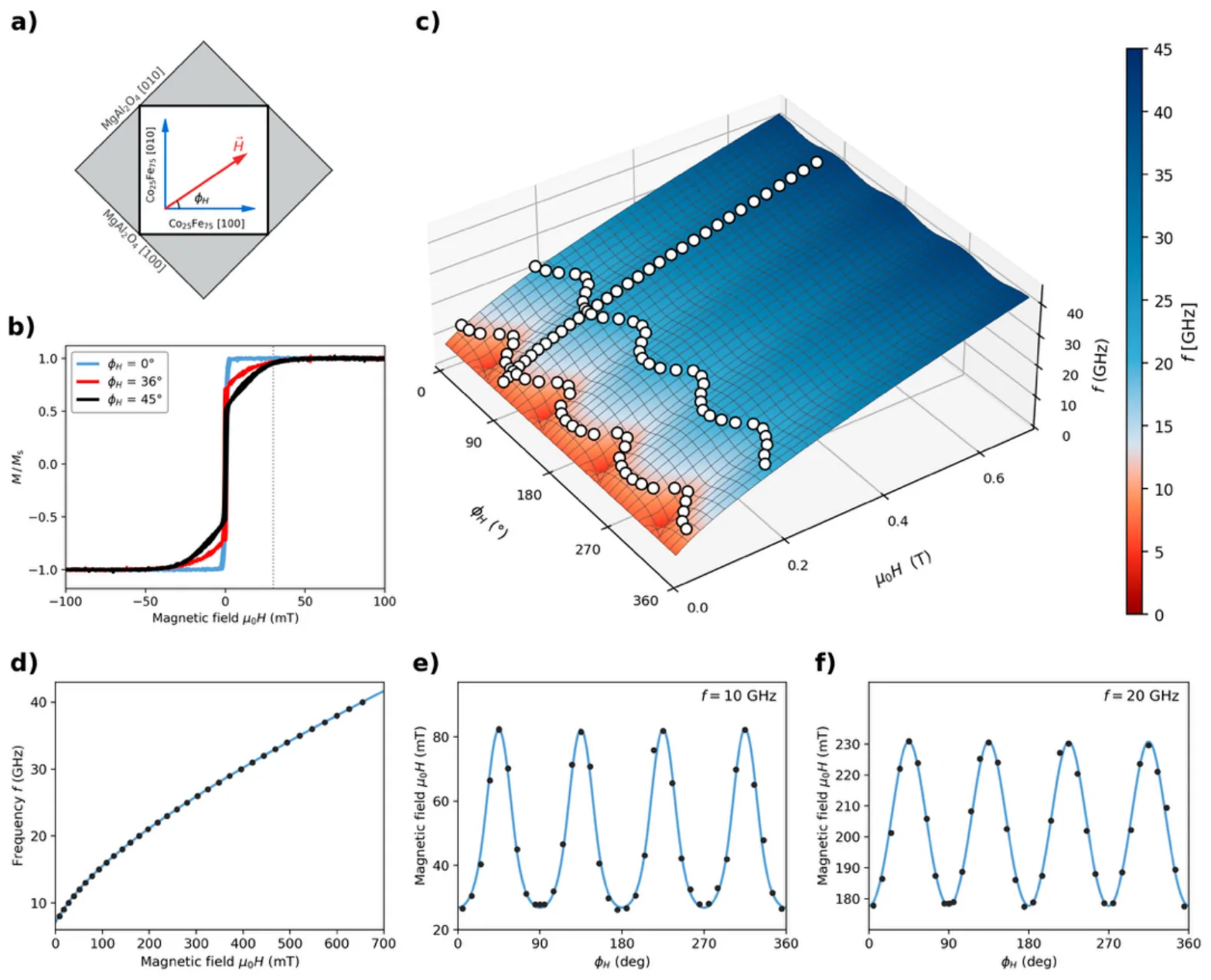
(**a**) Schematic illustration of the crystallographic orientation of the epitaxial Co_25_Fe_75_ film with respect to the MgAl_2_O_4_ (001) substrate. The in-plane magnetic field angle, $\phi_{H}$, is measured with respect to the Co_25_Fe_75_ [100] crystallographic direction. (**b**) In-plane magnetic hysteresis loops measured at $\phi_{H}=$ 0°, 36°, and 45°. (**c**) Combined broadband and angular-dependent VNA-FMR data in the high-frequency regime. The solid surface represents the fit obtained using Equation (6), while the white symbols denote the experimental data. (**d**–**f**) Cross-sections of the fitted surface shown in (**c**). (**d**) Frequency as a function of the resonance magnetic field for $\phi_{H}=$ 90°. (**e**,**f**) Angular dependence of the resonance field measured at fixed excitation frequencies of 10 and 20 GHz, respectively.

Here, $\mu_{0}$ is the vacuum permeability, $M_{s}$ is the saturation magnetization, and $H_{i}$ denotes the anisotropy fields. The coordinate system associated with the cubic anisotropy is shown in Figure 1a: $\phi_{H}=0{}^{\circ}$ corresponds to the magnetic field applied along the [100] crystallographic direction of the Co_25_Fe_75_ film, whereas $\phi_{H}=45{}^{\circ}$ corresponds to the field applied along the [110] direction.

Using the Smit–Beljers equation [29,30,31], the Kittel equation for an in-plane applied magnetic field ($\theta_{H}=\theta_{M}=90{}^{\circ}$) can be written as:

(6) $${\left(\frac{2\pi f}{\gamma \mu_{0}}\right)}^{2}=\left(H\cos\left(\phi_{H}-\phi_{M}\right)+H_{4}\cos4\phi_{M}\right)\cdot \left(H\cos\left(\phi_{H}-\phi_{M}\right)+\frac{1}{4}H_{4}\left(\cos4\phi_{M}+3\right)+M_{eff}\right),$$

where $\gamma =\mu_{B}g/\hbar$ is the gyromagnetic ratio, $\mu_{B}$ is the Bohr magneton, g is the spectroscopic *g*-factor, ℏ is the reduced Planck constant, f is the microwave frequency, H is the resonance magnetic field, and $M_{eff}=M_{s}-H_{2⊥}$ is the effective magnetization.

Equation (6) shows that the frequency f depends on the in-plane magnetic field direction, described by the angle $\phi_{H}$, and on the magnetization orientation, described by the azimuthal angle $\phi_{M}$. Determination of $\phi_{M}$ requires finding the global minimum of the free energy. For an in-plane applied magnetic field, the free energy simplifies to $F_{IP}=F({\theta_{M}=\theta }_{H}=90{}^{\circ})$:

(7) $$\frac{F_{IP}}{\mu_{0}M_{s}}=-H\cos\left(\phi_{H}-\phi_{M}\right)+\frac{1}{2}H_{4}\;{cos}^{2}\left(\phi_{M}\right)\;{sin}^{2}\left(\phi_{M}\right).$$

The equilibrium magnetization orientation can be determined using numerical techniques. Computationally, one may define a numerical function $\phi_{M}\left(H,\phi_{H},H_{4}\right)$ that takes $H,\phi_{H},H_{4}$ as input parameters and returns the value of $\phi_{M}$ for which $F_{IP}$ reaches its minimum. In the present work, such an implementation was developed independently in Python (using imported MATLAB routines) and in Wolfram Mathematica.

Substituting $\phi_{M}\left(H,\phi_{H},H_{4}\right)$ into Equation (6) yields a functional dependence of the form $f\left(H,\phi_{H},g,H_{4},M_{eff}\right)$. Conventionally, the determination of in-plane anisotropies and the effective magnetization from FMR experiments relies on angular measurements, in which the resonance field H is measured as a function of $\phi_{H}$ at a fixed microwave frequency [32]. However, this approach requires assuming a value of the *g*-factor [8,32], or alternatively performing additional measurements, preferably at high frequencies, where the influence of the *g*-factor on the $f\left(H\right)$ dependence becomes more pronounced [33].

In the present work, we propose an alternative approach in which angular-dependent and broadband VNA-FMR data are fitted simultaneously to the two-dimensional surface $f\left(H,\phi_{H}\right)$. This procedure enables the simultaneous determination of $g,H_{4}$ and $M_{eff}$. A practical advantage of this method is that standard fitting routines available in software environments such as MATLAB or Mathematica provide estimates of the standard errors of the fitted parameters straightforwardly. Both implementations tested in this work yielded identical fitting results. Although differences in the numerical implementation of $\phi_{M}\left(H,\phi_{H},H_{4}\right)$ affect computational efficiency, the fitting time for a dataset containing 107 experimental points remained below ten seconds.

## 4. Results and Discussion

### 4.1. Magnetization Reversal Curves

Figure 1b shows hysteresis loops measured at $\phi_{H}$ = 0° (along the [100] direction of Co_25_Fe_75_), $\phi_{H}$ = 45° (along the [110] direction), and at the intermediate angle of $\phi_{H}$ = 36°. For each magnetization reversal curve, a small linear diamagnetic contribution arising from the MgAl_2_O_4_ substrate and the glass sample holder was subtracted. At $\phi_{H}$ = 0°, the nearly square hysteresis loop indicates that the [100] is the in-plane easy magnetization axis of the Co_25_Fe_75_ film. In contrast, for $\phi_{H}$ = 45°, the magnetization saturates at an applied magnetic field of approximately 30 mT (dashed vertical line in Figure 1b), indicating that the [110] direction is the in-plane hard magnetization axis. This field is identified as the magnetocrystalline anisotropy field and is further discussed and confirmed by the FMR measurements presented below. Similarly, at $\phi_{H}$ = 36°, the magnetization reaches 0.965 $M_{s}$ at an applied field of 30 mT, indicating that the film is already close to magnetic saturation.

To determine the saturation magnetization of the Co_25_Fe_75_ film using the VSM technique, a permalloy reference sample was employed. The saturation magnetization, $M_{s}$, of the sample under investigation can be determined using:

(8) $$M_{s}=\frac{\epsilon }{\epsilon_{ref}}\frac{A_{ref}}{A}\frac{d_{ref}}{d}M_{s}^{ref}$$

where ϵ is the amplitude of the induced voltage at magnetic saturation, A is the film area, and d is the film thickness. The subscript “ref” denotes the corresponding parameters of the reference permalloy sample. The sample areas were determined from photographs taken on millimeter paper and analyzed using graphical software. The reference sample consisted of a 166 nm-thick permalloy film deposited by magnetron sputtering at room temperature onto a silicon substrate of approximately the same dimensions as the MgAl_2_O_4_ substrates (about 1 × 1 cm^2^). Under these deposition conditions, the permalloy film exhibited no measurable in-plane magnetic anisotropy, and owing to its relatively large thickness, interfacial and surface anisotropy contributions were assumed to be negligible. Therefore, we performed VNA-FMR measurements for the permalloy film, and using the Kittel equation, $2\pi f=\gamma \mu_{0}\sqrt{H\left(H+M_{s}\right)}$, we determined $g^{Py}$ = 2.1009 ± 0.0016, and $M_{s}^{Py}$ = 832 ± 2 kA/m. The root-mean-square value of the fitting residuals was 6.5 MHz.

Using Equation (8), the saturation magnetization of the Co_25_Fe_75_ film was determined to be $M_{s}$ = 1920 ± 170 kA/m ($\mu_{0}M_{s}$ = 2.41 ± 0.21 T). The estimated uncertainty of approximately ≈9% arises from the combined uncertainties of the parameters in Equation (8), with the dominant contribution originating from the uncertainty in the film volume. The obtained value is in good agreement with our previous measurements on polycrystalline Co_25_Fe_75_ films ($M_{s}$ = 2070 kA/m) [7] and with literature values ranging from 1863 to 1967 kA/m [1,2,4,5,14,15].

### 4.2. High Frequency, Field-Swept VNA-FMR Results

Figure 1c presents the combined VNA-FMR dataset, consisting of broadband measurements with the magnetic field applied along the in-plane easy magnetization axis ($\phi_{H}$ = 90°) and angular-dependent measurements performed at fixed excitation frequencies of 10 and 20 GHz. The fourfold symmetry of the resonance field as a function of $\phi_{H}$ is a clear signature of the cubic magnetocrystalline anisotropy and confirms the epitaxial growth of the Co_25_Fe_75_ film on the single-crystalline MgAl_2_O_4_ (001). Fitting the experimental data with the two-dimensional surface $f\left(H,\phi_{H}\right)$ defined by Equation (6) enabled the simultaneous determination of the g-factor, $H_{4}$, and $M_{eff}$. The root-mean-square value of the fitting residuals, defined as the difference between the experimental and calculated resonance frequencies, was 113 MHz, which is small compared with the measured frequency range of 8–40 GHz. To further demonstrate the quality of the fit, which may not be readily apparent from the three-dimensional representation in Figure 1c, the individual broadband and angular-dependent datasets together with the corresponding fitting curves are shown in the conventional form in Figure 1d–f. The good agreement between the experimental data and the model confirms the validity of the proposed fitting approach.

The fitted values of g, $H_{4}$, and $M_{eff}$ are summarized in Table 1. The obtained *g*-factor, g = 2.083 ± 0.017, agrees well with the previously reported value of 2.107 ± 0.071 for an epitaxial Co_25_Fe_75_ film grown on MgO (001) and capped with Cr [4]. Furthermore, the measured value was closer to that of bulk Fe (2.085 ± 0.003) than to bulk Co (2.139 ± 0.005) [2]. For polycrystalline Co_25_Fe_75_ films, out-of-plane FMR measurements yielded g = 2.104 ± 0.007 [2]. The slightly lower value obtained in the present work may be attributed to interfacial effects, similar to those previously reported for thin Ni-Fe films [34].

The fitting yielded a cubic magnetocrystalline anisotropy field of $\mu_{0}H_{4}$ = 28.25 ± 0.22 mT. The corresponding anisotropy constant is related to this field through the relation $K_{4}=\mu_{0}H_{4}M_{s}/2$. Using the experimentally determined saturation magnetization, we obtained $K_{4}$ = 27.1 ± 2.4 kJ/m^3^. These values are consistent with previous reports for epitaxial Co_25_Fe_75_ films with a thickness of approximately 7 nm, for which the cubic anisotropy field was reported to range from 27.0 and 32.5 mT [4,8]. As shown in Figure 1e,f, no significant contribution from an in-plane uniaxial anisotropy was observed, indicating that the in-plane anisotropy is dominated by the cubic term. This observation is consistent with the good lattice matching between Co_25_Fe_75_ and MgAl_2_O_4_ (lattice mismatch of approximately 0.4%) and agrees with previous reports [4,8]. It is worth noting that the small variations in the in-plane uniaxial anisotropy field (±1.5 mT) reported in Ref. [8] exhibited no systematic dependence on film thickness. This suggests that they may originate from a slight substrate miscut, i.e., a small deviation of the polished substrate surface from the ideal (001) crystallographic plane [35,36,37,38,39,40]. Therefore, the presence of a small in-plane uniaxial anisotropy in Co_25_Fe_75_/MgAl_2_O_4_ system may depend on the quality of the substrate surface and consequently vary from sample to sample.

The effective magnetization was determined to be $M_{eff}$ = 1655 ± 31 kA/m. It should be noted that in thin films, the effective magnetization is governed by the out-of-plane uniaxial anisotropy field, $H_{2⊥}$. This anisotropy may originate from several mechanisms, including interfacial (surface) anisotropy, strain-induced anisotropy, and growth-induced anisotropy. Consequently, $M_{eff}$ is sensitive both to the interfaces surrounding the Co_25_Fe_75_ layer (e.g., the substrate and capping layer) and to the deposition conditions affecting the epitaxial growth. The value obtained in this work is in good agreement with previous studies. For example, 7-nm thick Co_25_Fe_75_ films grown on MgAl_2_O_4_ exhibited effective magnetizations between 1520 and 1560 kA/m, whereas films deposited on MgO showed values between 1740 and 1790 kA/m [4,8]. In both studies [4,8], the films were capped with Cr to prevent oxidation, whereas in the present work, a 5 nm-thick Au capping layer was used. This difference in the adjacent interface may contribute to the observed variation in the effective magnetization.

### 4.3. Low Field, Frequency-Swept VNA-FMR Results

Following the modeling presented in Figure 1c, substantial variations in the resonance frequency were predicted for magnetic fields between 0 to 100 mT. In particular, the fourfold symmetry of the cubic magnetocrystalline anisotropy gives rise to four minima in the $f\left(H,\phi_{H}\right)$ surface, corresponding to the in-plane hard magnetization directions. The model further predicts a pronounced flattening of the $f\left(H\right)$ dependence at intermediate values of $\phi_{H}$, i.e., between the principal crystallographic directions (Figure 1c). To verify this prediction experimentally and to better understand its origin, frequency-swept VNA-FMR measurements were performed in the low-field regime.

**Figure 2:**
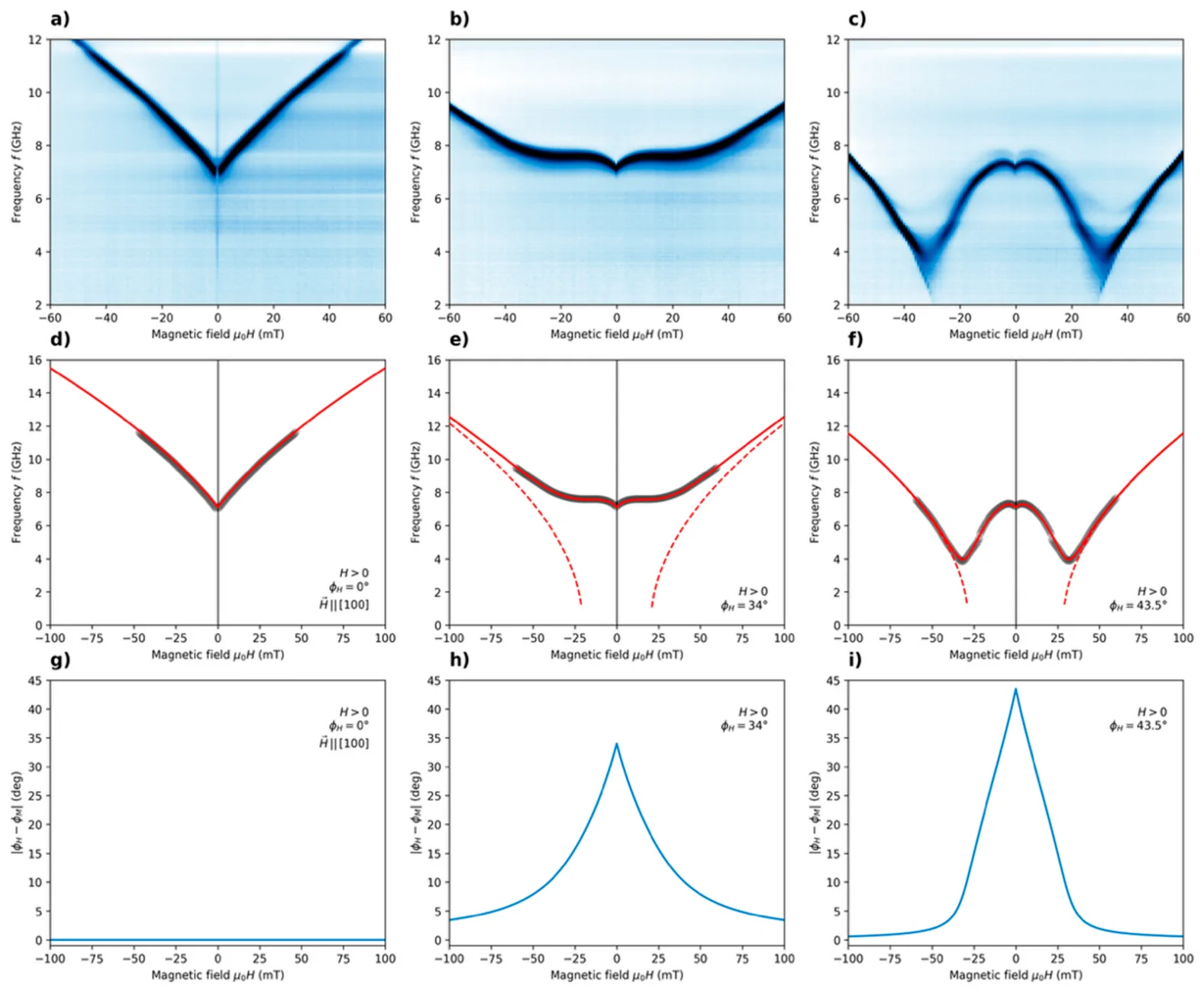
Frequency-swept VNA-FMR measurements and modeling in the low-field regime for the epitaxial Co_25_Fe_75_ film grown on MgAl_2_O_4_ (001). (**a**–**c**) Color-coded experimental VNA-FMR spectra measured at (**a**) $\phi_{H}=$ 0° ($\vec{H}∥[100]$); (**b**) $\phi_{H}=$ 34°; and (**c**) $\phi_{H}=$ 43.5°. In (**b**), the signal-to-noise ratio (SNR) was estimated as the ratio of the mean resonance peak amplitude to the standard deviation of the off-resonance background noise, yielding SNR ≈ 26.8. The resulting high SNR enabled reliable determination of the resonance position. (**d**–**f**) Calculated resonance dispersions $f\left(H\right)$ superimposed on the corresponding experimental data shown in (**a**–**c**). Solid lines represent calculations based on Equation (6) using g = 2.083, $\mu_{0}H_{4}=$ 28.3 mT, and $M_{eff}=$ 1650 kA/m. The dashed lines show the results of Equation (6) calculated under the assumption $\phi_{M}=\phi_{H}$. (**g**–**i**) Dependence of $\left|\phi_{H}-\phi_{M}\right|$ on the magnitude of the external magnetic field, illustrating the in-plane non-collinearity between $\vec{M}$ and $\vec{H}$.

Figure 2a–c shows the measured spectra for $\phi_{H}$ = 0° ($\vec{H}∥[100]$), $\phi_{H}$ = 34°, and at $\phi_{H}$ = 43.5° (close to the in-plane hard axis). The calculated resonance dispersions, shown as solid lines in Figure 2d–f and obtained using Equation (6), are in excellent agreement with the experimental data, thereby confirming the values of the g-factor, cubic anisotropy field, and effective magnetization determined from the high-frequency measurements.

At $\phi_{H}$ = 0°, the magnetic field is applied along the in-plane easy axis. Consequently, the magnetization remains parallel to the external field, and $\left|\phi_{H}-\phi_{M}\right|=0{}^{\circ}$ throughout the entire magnetic-field range (Figure 2g). In contrast, the modeling shows that at $\phi_{H}$ = 43.5°, magnetization gradually rotates away from the field direction toward the easy axis as the magnetic field decreases below approximately 30 mT (Figure 2i).

The most intriguing behavior was observed for $\phi_{H}$ = 34°, where a pronounced plateau appeared in the $f\left(H\right)$ dependence, extending up to approximately 30 mT. To gain further insight into this behavior, Equation (6) was evaluated under two different assumptions. The solid line in Figure 2e was calculated using the equilibrium magnetization direction obtained by minimizing the free energy, whereas the dashed line corresponds to the simplified assumption $\phi_{M}=\phi_{H}$. The substantial discrepancy between these two calculations demonstrates that the observed flattening is governed by the magnetization equilibrium condition. For magnetic fields above 100 mT, the angular difference $\left|\phi_{H}-\phi_{M}\right|$ remained below 4°. However, as the magnetic field decreased below 100 mT, $\left|\phi_{H}-\phi_{M}\right|$ increased gradually, eventually reaching 34° (Figure 2h). In other words, as the magnetic field decreases, the magnetization progressively rotates toward the nearest easy axis.

Importantly, this tilting of the magnetization had little effect on the intensity of the FMR absorption peak (Figure 2b), indicating that the magnetization undergoes coherent rotation over the entire investigated field range. This field-dependent rotation modifies the relative contributions of the Zeeman and cubic anisotropy terms to the FMR resonance condition (Equation (6)). Over a finite range of magnetic fields, these contributions partially compensate for each other, resulting in a region where the resonance frequency changes only weakly with magnetic field.

### 4.4. Flattening of Frequency vs. Magnetic Field Dependence

**Figure 3:**
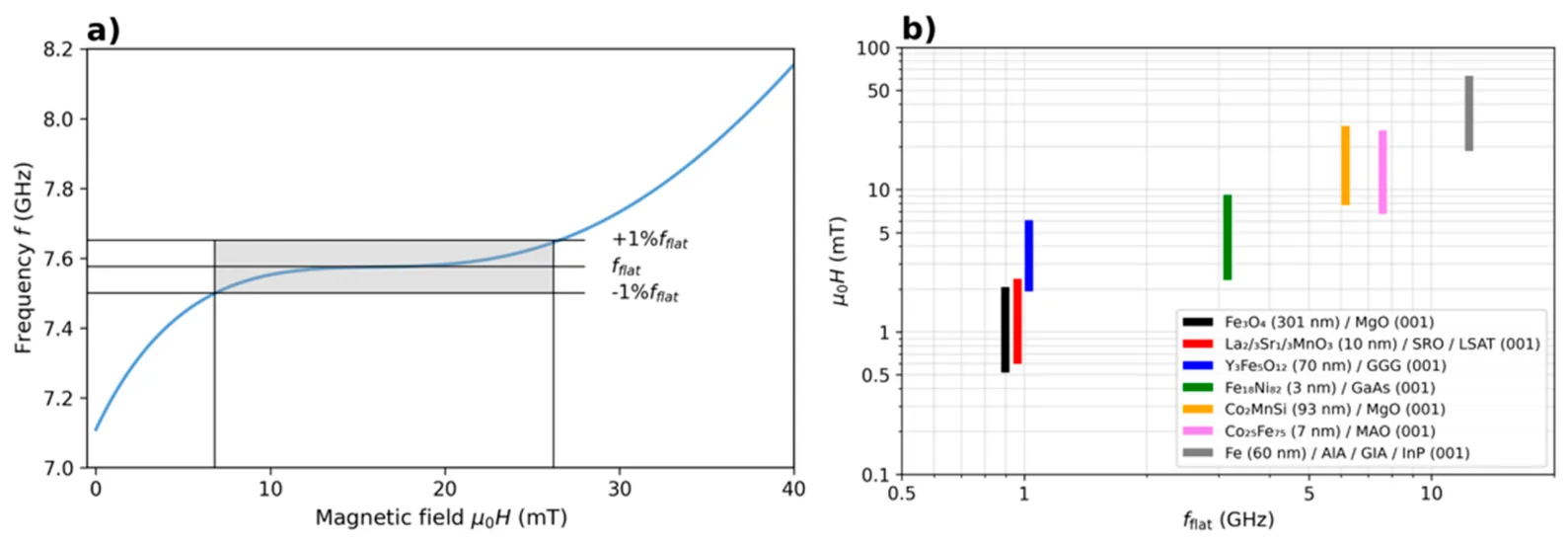
(**a**) Enlarged view of the resonance frequency as a function of the applied magnetic field for $\phi_{H}=$ 34° in the low-field regime for the epitaxial Co_25_Fe_75_ film studied in this work. The gray rectangle indicates the frequency plateau, defined as the range within ±1% of $f_{flat}$, together with the corresponding magnetic-field interval. (**b**) Magnetic-field intervals over which the resonance frequency remains nearly constant (within ±1% of $f_{flat}$) for different (001)-oriented epitaxial thin films.

Figure 3a shows an enlarged view of the resonance frequency as a function of the external magnetic field for $\phi_{H}$ = 34° in the epitaxial Co_25_Fe_75_ film investigated in this work. To quantify the observed frequency plateau, we defined the characteristic frequency $f_{flat}$ as the resonance frequency at the inflection point of the $f\left(H\right)$ dependence. The plateau was then defined as the frequency range within ±1% of $f_{flat}$, highlighted by the gray rectangle in Figure 3a. Using this criterion, we determined the corresponding magnetic-field interval over which the resonance frequency remains nearly constant. For the Co_25_Fe_75_ film, this interval extended from 6.8 to 26.2 mT at room temperature. Considering a ±0.5% tolerance, the interval was 8.6–23.4 mT, while for a ±2% tolerance, it extended from 4.6 to 29.4 mT.

To place these results in a broader context, we analyzed literature data for epitaxial thin films exhibiting dominating cubic magnetocrystalline anisotropy. The magnetic parameters of the selected materials are summarized in Table 2. Using the reported spectroscopic *g*-factor, the cubic anisotropy field, and the effective magnetization, we calculated the characteristic frequency $f_{flat}$ and the corresponding magnetic-field interval over which the resonance frequency remained within ±1% of $f_{flat}$. The calculated results are presented in Figure 3b.

For the considered materials, with effective magnetizations ranging from 127 to 1720 kA/m and cubic anisotropy fields between 2.2 and 69.8 mT, the characteristic frequencies $f_{flat}$ spanned from 0.9 to 12.35 GHz. More importantly, the corresponding magnetic-field intervals extended from 0.5 to 63 mT. These results demonstrate that by selecting an appropriate material, it is possible to realize frequency plateaus over a wide range of magnetic fields. Consequently, for applications requiring resonance frequencies that are insensitive to magnetic-field variations, both the operating frequency and the corresponding field interval can be tailored through an appropriate choice of material.

## 5. Conclusions

In summary, we investigated the dynamic magnetic properties of an epitaxial Co_25_Fe_75_ thin film grown on a single-crystalline MgAl_2_O_4_ (001) substrate. To analyze the VNA-FMR measurements, we employed the conventional Kittel equation together with a numerically implemented function for determining the equilibrium magnetization orientation. This approach enables the use of standard nonlinear fitting routines, providing straightforward access to parameter uncertainties and statistical measures of the fit. Moreover, the methodology can be generalized to include higher-order anisotropy terms and angle-dependent measurements involving both the azimuthal and polar directions, making it applicable to a broad range of magnetic thin-film systems.

From the VNA-FMR measurements, we determined an effective magnetization of 1655 ± 31 kA/m for the Au-capped Co_25_Fe_75_/MgAl_2_O_4_ film. The layer exhibited a pronounced fourfold symmetry of the resonance field, corresponding to a cubic magnetocrystalline anisotropy field of 28.25 ± 0.22 mT. At the intermediate in-plane field orientation of $\phi_{H}=$ 34°, i.e., between the principal crystallographic directions (0° and 45°), a pronounced plateau was observed in the resonance frequency as a function of the applied magnetic field. The observed behavior is fully explained within the Stoner–Wohlfarth framework through the equilibrium rotation of the magnetization. The frequency plateau extended over the magnetic-field interval from 6.8 to 26.2 mT at room temperature.

A survey of other epitaxial thin films exhibiting predominantly cubic magnetocrystalline anisotropy showed that by appropriate material selection, both the characteristic resonance frequency and the corresponding magnetic-field interval over which the frequency remains nearly constant can be widely tuned. For the materials considered in this work, the characteristic plateau frequencies spanned from 0.9 to 12.35 GHz, while the corresponding magnetic-field intervals over which the frequency remained in a nearly constant range from 0.5 to 63 mT.

From an application perspective, the stability of the resonance frequency over a broad magnetic-field interval provides a route toward microwave devices that are inherently less sensitive to fluctuations of the external magnetic field. Furthermore, the coexistence of a frequency plateau and a controllable non-collinearity between the magnetization and the applied field raises intriguing questions regarding spin-wave propagation in this regime. In particular, the possibility of steering spin-wave beam direction by varying only the magnitude of the bias magnetic field merits further theoretical and experimental investigation.

## Figures and Tables

**Table 1:** Magnetic properties of the Co_25_Fe_75_ film deposited on MgAl_2_O_4_ (001).

Au (5 nm)/Co_25_Fe_75_ (7 nm)/MgAl_2_O_4_ (001)		
Saturation magnetization	$M_{s}$ (kA/m)	1920 ± 170
Landé g-factor	g	2.083 ± 0.017
Effective magnetization	$M_{eff}$ (kA/m)	1655 ± 31
Cubic anisotropy field	$\mu_{0}H_{4}$ (mT)	28.25 ± 0.22
Cubic anisotropy constant	$K_{4}$ (kJ/m^3^)	27.1 ± 2.4

**Table 2:** Magnetic properties of selected (001)-oriented thin films: spectroscopic *g*-factor, cubic magnetocrystalline anisotropy field $H_{4}$, effective magnetization $M_{eff}$, calculated frequency $f_{flat}$ at $\phi_{H}=$ 34°, and the corresponding magnetic-field interval over which the resonance frequency remained within ±1% of $f_{flat}$. All referenced substrates were (001)-oriented.

Ref.	Film, Thickness	Buffer/Substrate	*g*	$\mu_{0}H_{4}$ (mT)	$M_{eff}$ **(kA/m)**	$f_{flat}$ (GHz)	Field Interval (mT)
[41]	Fe_3_O_4_ (301 nm)	MgO	2.14	2.20	285	0.897	0.52–2.08
[42]	La_2/3_Sr_1/3_MnO_3_ (10 nm)	SrRuO_3_/(LaAlO_3_)_0.3_(Sr_2_AlTaO_6_)_0.7_	2.00	2.50	330	0.962	0.60–2.38
[43]	Y_3_Fe_5_O_12_ (70 nm)	Gd_3_Ga_5_O_12_	2.01	6.95	127	1.026	1.94–6.12
[44]	Fe_18_Ni_82_ (3 nm)	GaAs	2.10	10.0	800	3.150	2.33–9.23
[45]	Co_2_MnSi (93 nm)	MgO	2.07	30.7	996	6.141	7.80–28.0
This work	Co_25_Fe_75_ (7 nm)	MgAl_2_O_4_	2.08	28.3	1650	7.570	6.80–26.2
[46]	Fe (60 nm)	Al_0.48_In_0.52_As/Ga_0.47_In_0.53_As/InP	2.09	69.8	1720	12.35	18.8–63.2

**Figure A1:**
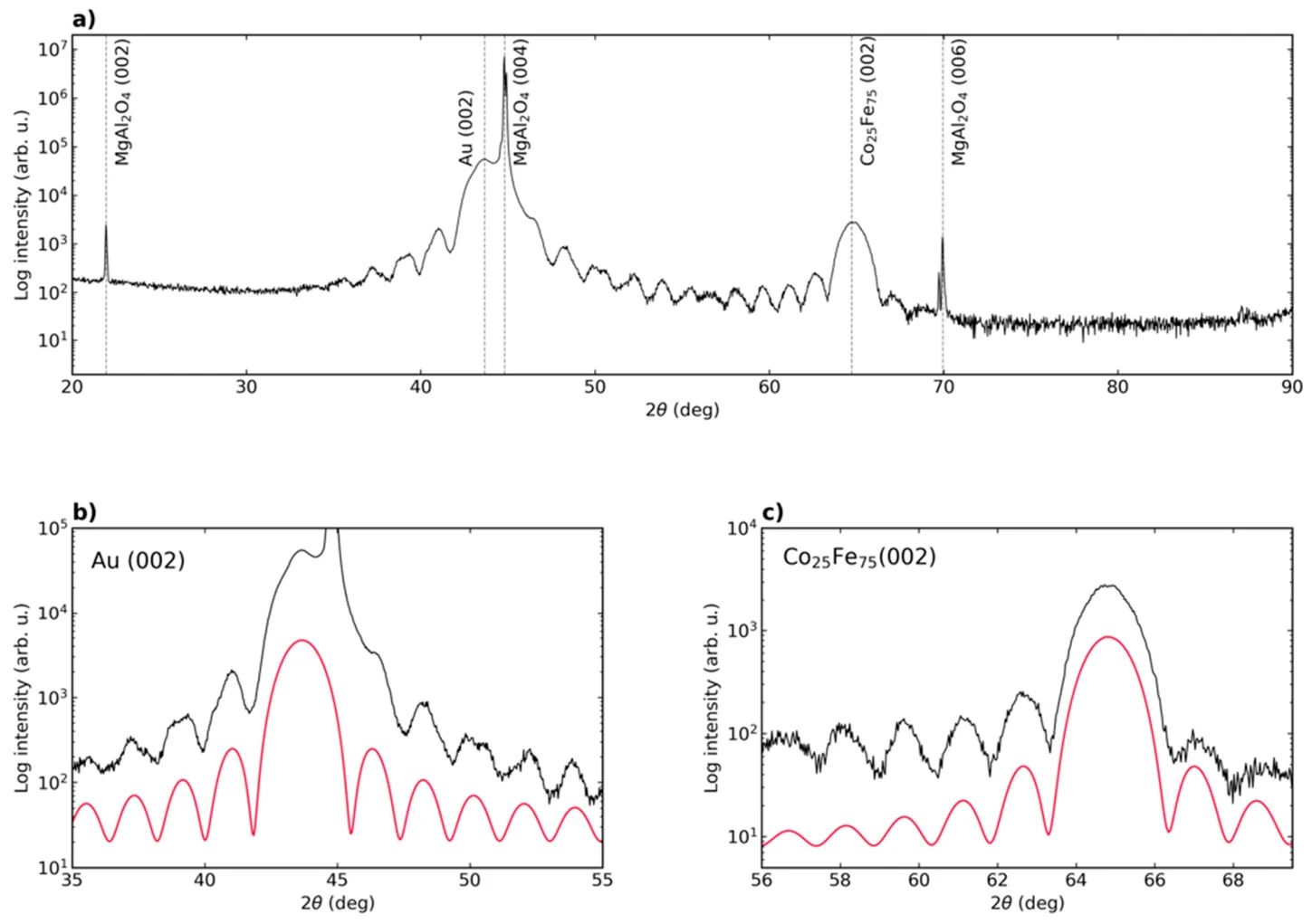
(**a**) X-ray diffraction pattern of the Au-capped Co_25_Fe_75_ film grown on the MgAl_2_O_4_ (001) substrate. (**b**,**c**) Enlarged views of the diffraction peaks corresponding to the Au (002) and MgAl_2_O_4_ (002) reflections, respectively. The red solid lines represent modeling of the Laue oscillations using Equation (A1).

**Figure A2:**
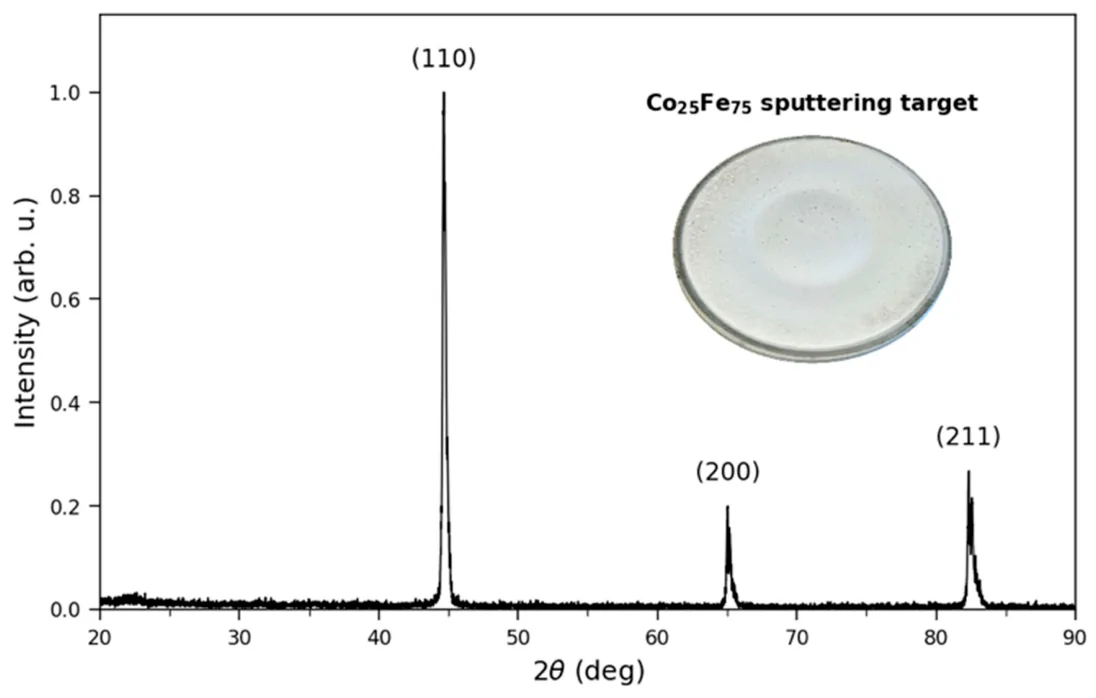
X-ray diffraction pattern of the polycrystalline Co_25_Fe_75_ sputtering target.

**Figure A3:**
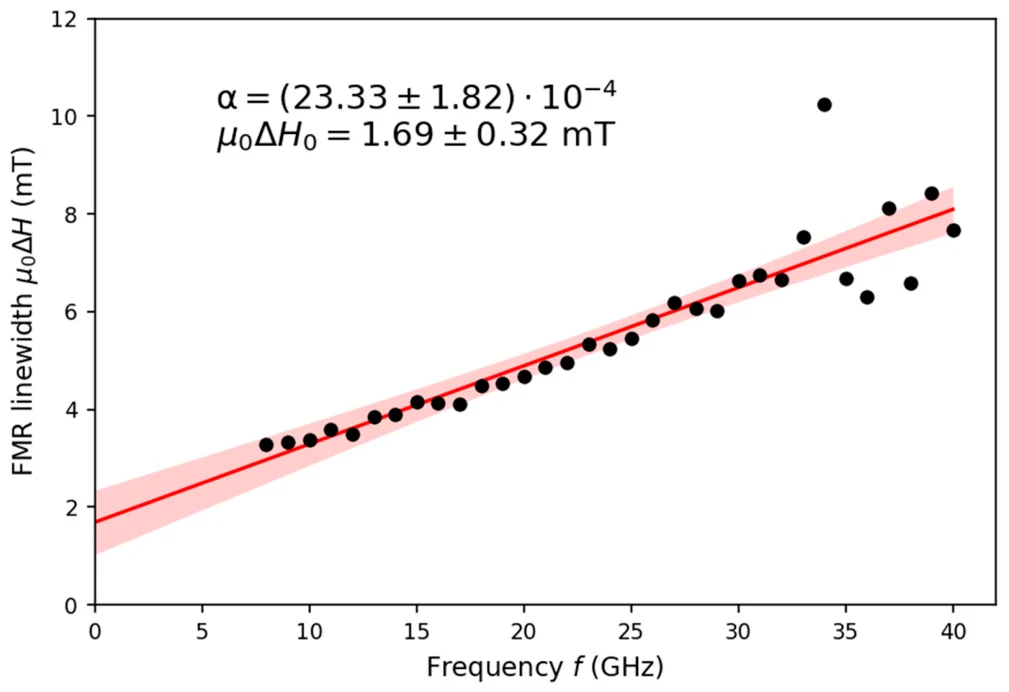
Resonance linewidth as a function of frequency for the magnetic field applied along the in-plane easy magnetization axis of the Co_25_Fe_75_ film ($\phi_{H}$ = 90°). The red solid line represents the linear fit to the experimental data [57,58,59,60], and the light-red shaded region denotes the corresponding 95% confidence band, illustrating the uncertainty associated with the fitted parameters. The inset summarizes the determined values of the Gilbert damping parameter α, and the inhomogeneous linewidth broadening $\mu_{0}{∆H}_{0}$. The obtained Gilbert damping parameter is consistent with previously reported values ranging from 15 × 10^−4^ to 53.5 × 10^−4^ [1,6,7,15,26].

## Data Availability

The original contributions presented in this study are included in the article. Further inquiries can be directed to the corresponding authors.

## Appendix A

To investigate the structural properties of the sample, X-ray diffraction (XRD) measurements were performed using an X’Pert PRO diffractometer [47,48,49,50,51] (Malvern Panalytical, Worcestershire, UK). The resulting diffraction pattern is shown in Figure A1a. The most intense peaks, observed at 2θ = 44.790° (Cu Kα_1_) and 2θ = 44.906° (Cu Kα_2_), corresponded to the MgAl_2_O_4_ (004) reflection. Using Bragg’s law, the lattice parameter of the substrate was determined to be $a_{MAO}$ = 0.8087 nm (Table A1), in good agreement with literature values of 0.8080–0.8084 nm [52]. Additional weak peaks were observed at 2θ = 21.964° and 2θ = 69.708°, corresponding to the basis-forbidden MgAl_2_O_4_ (002) and (006) reflections, respectively. These reflections originated from multiple diffraction (the Renninger effect), as previously reported [22,53,54].

In the vicinity of the substrate (004) reflection, the Au (002) peak from the capping layer was observed at 2θ = 43.631°. This corresponded to an out-of-plane lattice parameter of $a_{Au}$ = 0.4146 nm, which was approximately 1.8% larger than the bulk value of 0.4073 nm [55]. The observed expansion indicates a small tetragonal distortion of the Au unit cell. No additional Au reflections were detected, indicating a strong crystallographic texture of the capping layer.

Similarly, the Co_25_Fe_75_ (002) reflection was observed at 2θ = 64.762°. The corresponding out-of-plane lattice parameter was $a_{CoFe}$ = 0.2877 nm, which was in excellent agreement with the previously reported values for 7 nm-thick Co_25_Fe_75_ films (0.2873–0.2876 nm) [8]. Compared with the bulk value of 0.28664 nm for Co_25_Fe_75_ (see Appendix B), the lattice parameter was increased by approximately 0.37%. This result confirms the excellent lattice matching between Co_25_Fe_75_ and MgAl_2_O_4_ through the supercell relation $\sqrt{2}a_{MAO}\approx 4a_{CoFe}$, resulting in only a small lattice mismatch, and consequently, only a slight tetragonal distortion of the Co_25_Fe_75_ unit cell.

Both the Au capping layer and the Co_25_Fe_75_ film exhibited pronounced Laue oscillations in the vicinity of their respective diffraction peaks (Figure A1b,c), demonstrating the high crystalline quality of the layers. The presence of Laue oscillations indicates atomically smooth interfaces, uniform film thickness, and a low density of structural defects [56]. The positions of the satellite reflections depend sensitively on the film thickness and can be described using the Laue interference function [56]:

(A1) $$I\sim \frac{{sin}^{2}\left(q_{1}d/2\right)}{{sin}^{2}\left(q_{1}a_{z}/2\right)}+\frac{1}{2}\frac{{sin}^{2}\left(q_{2}d/2\right)}{{sin}^{2}\left(q_{2}a_{z}/2\right)},$$

(A2) $$q_{1,2}=\frac{4\pi }{\lambda_{1,2}}sin\left(\frac{2\theta }{2}\right),$$

where $a_{z}$ is the out-of-plane lattice parameter, d is the film thickness, $\lambda_{1}$ = 1.540598 Å and $\lambda_{2}$ = 1.544426 Å are the wavelengths of the Cu Kα_1_ and Cu Kα_2_ radiation, respectively. The modeling using Equation (A1) is shown as red solid lines in Figure A1b,c. From the analysis, the thicknesses of the Au and Co_25_Fe_75_ layers were determined to be 5.18 nm and 6.90 nm, respectively, in excellent agreement with the nominal thicknesses targeted during film deposition.

**Table A1:** Structural parameters of the Au-capped Co_25_Fe_75_ film grown on the MgAl_2_O_4_ (001) substrate, determined from the X-ray diffraction measurements. Here, (*h k l*) denotes the Miller indices, 2θ is the total scattering angle corresponding to the Cu Kα_1_ radiation ($\lambda_{1}=$ 1.540598 Å), $a_{z}$ is the out-of-plane lattice parameter, and d is the film thickness.

Material	*h k l*	2θ (deg)	$a_{z}$ (nm)	d (nm)
MgAl_2_O_4_	0 0 4	44.790	0.8087	---
Co_25_Fe_75_	0 0 2	64.762	0.2877	6.90
Au	0 0 2	43.631	0.4146	5.18

## Appendix B

To determine the lattice parameter of bulk Co_25_Fe_75_, XRD measurements were performed on the stoichiometric polycrystalline sputtering target used for film deposition. The measurements were carried out using a Seifert 3003 TT diffractometer. The resulting diffraction pattern is shown in Figure A2. Three diffraction peaks corresponding to the (110), (200), and (211) crystallographic planes were observed. Their positions are summarized in Table A2. Using the relation:

(A3) $$\sqrt{h^{2}+k^{2}+l^{2}}=2\frac{a_{CoFe}}{\lambda_{1}}sin\left(\frac{2\theta }{2}\right)$$

the lattice parameter of bulk Co_25_Fe_75_ was determined to be 0.28664 nm. The average crystallite size was estimated using the Scherrer equation, taking into account the instrumental broadening of the diffraction peaks. The resulting crystallite size was found to be approximately 89 nm.

**Table A2:** Miller indices (*h k l*), measured 2θ values, and relative intensities $I/I_{max}$ of the diffraction peaks of the polycrystalline Co_25_Fe_75_, obtained using Cu Kα_1_ radiation ($\lambda_{1}=$ 1.540598 Å).

*h k l*	2θ (deg)	$I/I_{max}$
1 1 0	44.664	100%
2 0 0	65.030	20%
2 1 1	82.332	26%
